# Comparison and analysis of alveolar bone structure characteristics in skeletal Class II and Class III malocclusion in the mandibular incisor region

**DOI:** 10.1097/MD.0000000000040184

**Published:** 2024-11-29

**Authors:** Yunqing Chen, Binbin Zhao

**Affiliations:** a Department of Orthodontics, Affiliated Dongyang Hospital of Wenzhou Medical University, Dongyang, Zhejiang, China; b Department of Orthodontics, Dongyang People’s Hospital, Dongyang, Zhejiang, China.

**Keywords:** Class II, Class III, malocclusion, mandibular incisor alveolar bone, skeletal

## Abstract

The aim of this study was to explore the differences in alveolar bone structure characteristics in skeletal class II and class III malocclusion in the mandibular incisor region. From January 2021 to November 2023, 100 cases of skeletal malocclusion patients were selected from our hospital, including 56 cases of skeletal Class II malocclusion and 44 cases of skeletal Class III malocclusion. The alveolar bone structure characteristics in the mandibular incisor region of skeletal class II and class III malocclusion patients are compared. The labial alveolar bone attachment height and lingual alveolar bone attachment height were compared between patients with skeletal Class II and Class III malocclusion (*P* < .05). The labial alveolar bone thickness at the root apex in patients with skeletal Class II malocclusion was (3.96 ± 0.63) mm, which was higher than that in Class III malocclusion patients (*P* < .05). In patients with skeletal Class II malocclusion, the cementoenamel junction was 2, 4, 6 mm below, and at the root apex, the alveolar bone thickness was (0.19 ± 0.05) mm, (0.93 ± 0.10) mm, (2.10 ± 0.10) mm, and (12.26 ± 2.10) mm, respectively, which was higher than that in Class III malocclusion patients (*P* < .05). In patients with skeletal Class II malocclusion, the labial side alveolar bone area at the root apex was (3.89 ± 0.72) mm^2^, which was higher than in Class III malocclusion patients (*P* < .05). On the lingual side, the alveolar bone thickness below cementoenamel junction at 6 mm and at the root apex was (1.95 ± 0.45) mm^2^ and (1.92 ± 0.51) mm^2^, respectively, which were higher than in Class III malocclusion patients (*P* < .05). Compared to skeletal Class II malocclusion, in skeletal Class III malocclusion, the alveolar bone thickness in the mandibular incisor area is thinner and narrower. This should be given special attention during orthodontic treatment.

## 1. Introduction

Class II and Class III skeletal malocclusions are common oral health conditions in clinical practice. Patients often exhibit morphological changes in the midface region, such as midfacial concavity, which significantly impacts both oral function and facial aesthetics.^[1,2]^ The alveolar bone is a crucial component of the lower jawbone, consisting of hard bone plates, periodontal ligaments, and tooth root components, which together form the bone tissue surrounding the gums.^[3]^ According to the study,^[4]^ individuals with different types of skeletal malocclusions exhibit distinct skeletal muscle statuses and bite forces, resulting in certain differences in alveolar bone morphology among patients with various types of skeletal malocclusions.^[5,6]^ Orthodontic-orthognathic combined treatment is a common method for treating skeletal Class II and Class III malocclusions. During the procedures, it is necessary to move the mandibular incisors either toward the lips or the tongue. The reconstruction of alveolar bone can be influenced by the orthodontic force application method, as well as the balance of lip and tongue muscles. Additionally, alveolar bone morphology plays a role in this process.^[7]^ Therefore, it is important to ascertain the patient’s alveolar bone structure characteristics before implementing treatment. Clinical observations have revealed that in patients with skeletal Class II and Class III malocclusions, the compensatory lingual inclination of the mandibular incisors can result in thinner labial alveolar bone plates compared to patients with skeletal Class I malocclusions. This can lead to root resorption, bone fenestration, gingival recession, and periodontal issues.^[8,9]^ However, there is limited research on the differences in alveolar bone structure characteristics in the mandibular incisor area between skeletal Class II and Class III malocclusions. We hypothesize that there are structural differences between Class II and Class III malocclusion alveolar bone in the mandibular incisor region, which may have guiding significance for clinical treatment. In order to further enhance the assessment and correction capabilities for skeletal malocclusions, this study aims to investigate the issues mentioned above.

## 2. Materials and methods

### 2.1. General data

This study was approved by the Ethics Committee of Dongyang People’s Hospital (2024-1107-K047). Hundred patients with skeletal malocclusion who visited our hospital from January 2021 to November 2023 were selected. Among them, there were 56 patients with skeletal Class II malocclusion and 44 patients with skeletal Class III malocclusion. Clinical data of patients with skeletal Class II and Class III malocclusions were compared (*P* > .05; Table 1). Inclusion criteria: the diagnosis meets the criteria of “contemporary orthodontics”^[10]^; skeletal class II and III malocclusion; adult patients; first visit; informed consent of patients. Exclusion criteria: asymmetric facial deformity; there are impacted teeth or congenital missing teeth in the anterior teeth area; temporomandibular joint disease; patients with a history of orthodontic treatment.

**Table 1:** Comparison of clinical data of patients with skeletal Class II and Class III malocclusions.

Group	Cases	Gender		Age (yr)	Body mass index (kg/m^2^)
		Male	Female		
Class II	56	27 (48.21)	29 (51.79)	24.45 ± 2.61	22.12 ± 2.14
Class III	44	20 (45.45)	24 (54.55)	25.10 ± 2.48	22.25 ± 2.06
t/c2		0.075		−1.263	−0.307
*P*		.784		.209	.760

### 2.2. Check methods

A Promax 3D CBCT scanner (Shanghai Bestman Medical Instrument Co., Ltd.) was used to capture lateral skull radiographs. The patient’s head was immobilized with both eyes looking straight ahead. The imaging parameters were set as follows: voltage 85 kV, current 21 mA, slice thickness 150 μm, scanning field height 150 mm, width 150 mm, exposure time 20 seconds, and radiation dose 0.029 smV. The acquired images were saved in DICOM format, and a region of interest was placed on the anterior teeth area in the horizontal plane. The Galileo Viewer software provided by the machine was used to measure relevant parameters of the alveolar bone height in the sagittal direction. These parameters included labial alveolar bone attachment height (LAH), lingual alveolar bone attachment height (LPH), alveolar bone thickness, labial and lingual alveolar bone thickness at 2, 4, 6 mm below the cementoenamel junction (CEJ), and at the root apex. Additionally, the labial alveolar bone area at the root apex and the lingual alveolar bone area at 6 mm below CEJ and at the root apex in the lower anterior teeth area were measured.

### 2.3. Statistical processing

The data were analyzed by SPSS22.0 software. The measurement data included age, body mass index, etc. The data were expressed as ($\bar{\chi }\pm s$), and the differences between groups were analyzed by *t* test. Enumeration data include gender, etc., data are expressed as n (%), χ^2^ test analysis of differences between groups; statistical significance standard: *P* < .05.

## 3. Results

### 3.1. Comparison of alveolar bone height in lower incisor area

The LAH and LPH of patients with skeletal Class II and Class III malocclusions were compared (*P* < .05; Table 2).

**Table 2:** The height of alveolar bone in lower incisor area of skeletal class II and class III malocclusion.

Groups	Cases	LAH (mm)	LPH (mm)
Class II	56	1.81 ± 0.32	1.85 ± 0.35
Class III	44	1.90 ± 0.40	1.93 ± 0.37
t		−1.250	−1.106
*P*		.214	.271

LAH = labial alveolar bone attachment height, LPH = lingual alveolar bone attachment height.

### 3.2. Comparison of alveolar bone thickness in lower incisor area

The buccal root apex alveolar bone thickness in patients with skeletal Class II malocclusion was higher than that in patients with skeletal Class III malocclusion (*P* < .05). Moreover, in patients with skeletal Class II malocclusion, the alveolar bone thickness at 2, 4, 6 mm below the CEJ on the lingual side, and at the root apex was higher than that in patients with skeletal Class III malocclusion (*P* < .05; Table 3).

**Table 3:** Comparison of alveolar bone thickness in the lower incisor area of skeletal class II and class III malocclusion.

Groups	Cases	Alveolar bone thickness (mm)			
		2 mm below CEJ	4 mm below CEJ	6 mm below CEJ	Apex of the root
Labial					
Class II	56	0.20 ± 0.07	0.86 ± 0.17	0.92 ± 0.11	3.96 ± 0.63
Class III	44	0.18 ± 0.06	0.92 ± 0.15	0.90 ± 0.14	2.78 ± 0.70
t		1.509	−1.844	0.800	8.853
* P*		.135	.068	.426	<.001
Lingual side					
Class II	56	0.19 ± 0.05	0.93 ± 0.10	2.10 ± 0.10	12.26 ± 2.10
Class III	44	0.05 ± 0.01	0.61 ± 0.11	1.75 ± 0.13	9.10 ± 1.65
t		18.269	15.200	15.221	8.188
* P*		<.001	<.001	<.001	<.001

CEJ = cementoenamel junction.

### 3.3. Comparison of alveolar bone area in lower incisor area

In patients with skeletal Class II malocclusion, the buccal root apex alveolar bone area was higher than that in patients with skeletal Class III malocclusion (*P* < .05). Additionally, in patients with skeletal Class II malocclusion, the lingual side alveolar bone area at 6 mm below the CEJ and at the root apex was higher than that in patients with skeletal Class III malocclusion (*P* < .05; Table 4).

**Table 4:** Comparison of alveolar bone area in the lower incisor area of skeletal class II and class III malocclusion.

Groups	Cases	Alveolar bone area (mm^2^)			
		2 mm below CEJ	4 mm below CEJ	6 mm below CEJ	Apex of the root
Labial					
* *Class II	56	0.17 ± 0.05	1.12 ± 0.10	1.02 ± 0.12	3.89 ± 0.72
* *Class III	44	0.16 ± 0.07	1.08 ± 0.12	1.00 ± 0.13	2.51 ± 0.61
*t*		0.833	1.818	0.797	10.164
* P*		.407	.072	.427	<.001
Lingual side					
* *Class II	56	0.22 ± 0.06	0.90 ± 0.14	1.95 ± 0.45	1.92 ± 0.51
* *Class III	44	0.20 ± 0.07	0.85 ± 0.16	1.28 ± 0.30	1.30 ± 0.43
*t*		1.537	1.665	8.499	6.458
* P*		.127	.099	<.001	<.001

CEJ = cementoenamel junction.

## 4. Discussion

Skeletal Class II and Class III malocclusions can significantly affect a patient’s oral function and facial aesthetics. Orthodontic treatment is the primary approach for managing these conditions,^[11]^ often involving the movement or tilting of the lower incisors in the labial or lingual direction. Successful treatment requires moving the mandibular incisors within the alveolar bone without encroaching upon the cortical bone.^[12]^ However, certain alveolar bone structural configurations may not be conducive to orthodontic treatment, and variations in the characteristics of the anterior mandibular alveolar bone among different types of malocclusion can pose challenges in clinical diagnosis and treatment.

Therefore, the purpose of this study was to analyze the differences in the structural characteristics of the alveolar bone in the lower incisor area between skeletal Class II and Class III malocclusions, aiming to provide a basis for clinical diagnosis and treatment. Cone-beam computed tomography (CBCT) technology effectively avoids the overlap and interference of adjacent tissues through real-time image display software, allowing for more accurate and detailed visualization of the alveolar bone structure.^[13]^ The results of this study showed significant differences in the labial alveolar bone height (LAH) and lingual alveolar bone height (LPH) between patients with skeletal Class II and Class III malocclusions (*P* < .05). In the normal anterior teeth area, LAH and LPH refer to the distances from the CEJ to the alveolar ridge on the labial and lingual sides, respectively. A larger numerical value indicates more significant resorption of the alveolar bone. Our findings exhibited some discrepancies compared to previous research, which may be attributed to differences in the inclusion criteria or variations in sample sizes. Additionally, the thickness of the alveolar bone is related to the variability of the mandibular symphysis (chin joint), but this aspect was not investigated in our study.

This study demonstrated that patients with skeletal Class II malocclusion have a thicker alveolar bone at the buccal (lip side) root apex area compared to those with skeletal Class III malocclusion. Additionally, in patients with skeletal Class II malocclusion, the alveolar bone thickness on the lingual (tongue-side)—measured at the enamel-dentin junction and at positions 2, 4, and 6 mm below the CEJ, as well as at the root apex—was significantly greater than in those with skeletal Class III malocclusion (*P* < .05). These findings suggest that compared to skeletal Class II malocclusion, the alveolar bone thickness in the mandibular anterior region is thinner in skeletal Class III malocclusion. Previous studies have found that patients with a high-angle facial profile tend to have thinner alveolar bone. Some researchers have observed that in many cases of high-angle or Class III malocclusion, the alveolar bone in the lower anterior region is narrow in shape.^[14]^ Moreover, other researchers have used CBCT analysis to examine the alveolar bone morphology in the lower anterior region of adults with different vertical facial patterns and found that the high-angle group had reduced bone thickness.^[15]^ The results of our study are similar to these previous findings. In skeletal malocclusions, individuals with skeletal Class II malocclusion often exhibit mandibular retrusion, while those with skeletal Class III malocclusion commonly present with maxillary protrusion. The reduced alveolar bone height in skeletal Class III malocclusion may be attributed to the lack of restraint from the upper anterior teeth, allowing for greater physiological upward movement of the lower anterior teeth. Additionally, limited functional stimulation and growth constraints on the mandibular arch contribute to this phenomenon. Furthermore, these results may also be a consequence of compensatory mechanisms in the craniofacial complex and the influence of functional stimuli.^[16]^

The occlusal function of the lower anterior teeth in patients with skeletal Class III malocclusion is reduced compared to those with Class II malocclusion, which can impact alveolar bone thickness and is related to the balance between functional stimulation and bone deposition. Our study’s findings indicated that patients with skeletal Class II malocclusion have a greater alveolar bone area at the labial root apex compared to those with skeletal Class III malocclusion. Additionally, in patients with skeletal Class II malocclusion, the alveolar bone thickness measured 6 mm below the CEJ and at the root apex is significantly greater than in patients with skeletal Class III malocclusion (*P* < .05). Patients with skeletal Class II malocclusion also exhibit greater labial and lingual inclination of the lower anterior teeth and a larger range of tooth root movement within the alveolar bone.^[17]^

In patients with skeletal Class III malocclusion, the lower anterior teeth area tends to be narrower, and the anterior teeth often adopt an edge-to-edge bite position, leading to malocclusion. This condition can also impede the development of the lower alveolar bone. Additionally, in skeletal Class III malocclusion, the lower anterior teeth frequently lack contact with the upper teeth, resulting in a shallower overbite compared to Class II malocclusion.^[18]^ However, the alveolar bone does not necessarily remodel as the anterior teeth shift forward, which explains why the alveolar bone thickness on the lingual side at 6 mm below the CEJ and at the root apex tends to be relatively narrow in patients with skeletal Class III malocclusion. These patients not only exhibit thinner alveolar bone in the root apex area compared to those with skeletal Class II malocclusion but also experience inadequate muscular forces within the maxillomandibular system. This results in weaker masticatory muscle strength, mandibular retrusion, insufficient development of bone mass in the lower anterior teeth area, and distinct characteristics compared to patients with skeletal Class II malocclusion.^[19,20]^

The 2 types of malocclusion, skeletal Class II and Class III, exhibit differences in bone density and malocclusion characteristics. Specifically, in patients with skeletal Class III malocclusion, the supporting area of the alveolar bone on the lip side near the root apex is smaller than in patients with skeletal Class II malocclusion. This study uses the measurement of the area of the alveolar bone on the labial and lingual sides of the tooth roots as an alternative to traditional linear measurements. This approach provides a more intuitive reflection of the overall characteristics of the alveolar bone.

Based on the research results mentioned above, it is important in clinical diagnosis and treatment to pay close attention to the alveolar bone morphology in the lower anterior teeth area of patients with skeletal Class III malocclusion. Careful consideration should be given to the selection of treatment methods and preventive measures to minimize iatrogenic adverse outcomes and achieve stable and aesthetically pleasing treatment goals. This includes a thorough evaluation of the alveolar bone structure and density when planning orthodontic or surgical interventions for patients with skeletal Class III malocclusion to ensure the best possible outcomes and long-term stability. For patients with skeletal Class II malocclusion, the greater alveolar bone thickness permits a wider range of orthodontic movements, reducing the risk of compromising bone integrity. Therefore, more substantial tooth movements, such as proclination or retraction of the lower anterior teeth, can be safely performed. In contrast, for patients with skeletal Class III malocclusion, the reduced alveolar bone thickness necessitates careful treatment planning to prevent adverse effects like bone dehiscence or gingival recession. Tooth movement should be limited, minimizing the degree of proclination or retraction of the lower anterior teeth to ensure that movements occur within the safe confines of the alveolar bone.

The study still has some limitations. This study is a retrospective study with selection bias and confounding bias. Secondly, the sample size of patients is small. Although this does not affect the accuracy of this study, the results of a larger sample size are more convincing.

In summary, compared with skeletal class II malocclusion, the alveolar bone thickness in the lower incisor area of skeletal class III malocclusion is thinner and narrower, which should be paid attention to in orthodontic treatment. Skeletal class II malocclusion can safely perform more substantial dental movements, such as the forward tilt or retraction of the front and lower teeth. For patients with skeletal class III malocclusion, tooth movement should be restricted.

## Author contributions

**Conceptualization, data curation, formal analysis, investigation, methodology, writing—original draft, writing—review & editing:** Yunqing Chen, Binbin Zhao.

**Supervision:** Binbin Zhao.
